# What factors explain the association between socioeconomic deprivation and reduced likelihood of live-donor kidney transplantation? A questionnaire-based pilot case-control study

**DOI:** 10.1136/bmjopen-2016-012132

**Published:** 2016-06-09

**Authors:** Phillippa K Bailey, Charles RV Tomson, Yoav Ben-Shlomo

**Affiliations:** 1 School of Social and Community Medicine, University of Bristol, Bristol, UK; 2 Freeman Hospital, 6uu Newcastle upon Tyne Hospitals NHS Foundation Trust, Newcastle upon Tyne, UK

**Keywords:** Live-donor kidney transplantation, Socioeconomic inequities, Pilot study, TRANSPLANT MEDICINE

## Abstract

**Objectives:**

Socioeconomically deprived individuals with renal disease are less likely to receive a live-donor kidney transplant (LDKT) than less deprived individuals. This study aimed to develop and pilot a questionnaire designed to determine what factors explain this association.

**Design:**

Questionnaire development and a pilot case–control study. Primary aims were to develop and evaluate a questionnaire, assess response rates, and to generate data to inform full-scale study design.

**Setting:**

A UK tertiary renal referral hospital and transplant centre.

**Participants:**

Invited participants comprised 30 LDKT recipients (cases) and 30 deceased-donor kidney transplant (DDKT) recipients (controls). Stratified random sampling was used to select cases and controls from all adults who had been transplanted at Southmead Hospital North Bristol National Health Service Trust, between 1 August 2007 and 31 July 2013.

**Methods:**

Participants were posted questionnaires that were accompanied by an invitation letter from the renal consultant responsible for their care, and a patient information leaflet. Non-responders were sent a second questionnaire after 4–6 weeks. Data were extracted from returned questionnaires, and entered onto a Research Electronic Data Capture (REDCap) database.

**Results:**

63% (n=38) of those invited returned questionnaires. 16 (42%) declined to answer the question on income. 58% of participants had not asked any of their potential donors to consider living kidney donation (52% LDKT vs 65% DDKT, p=0.44). There was some evidence of a difference between the R3K-T knowledge score for recipients of LDKTs (mean 6.7, SD 1.8) and for recipients of DDKTs (mean 4.9, SD 2.1), p=0.008. Variables’ distribution for the exposure variables of interest was determined.

**Conclusions:**

Findings from this study will inform a sample size calculation for a full-scale study. The findings of the full-scale case–control study will help us better understand how socioeconomic deprivation is related to the type of transplant an individual receives. This understanding will help us to design and appropriately tailor an intervention to reduce inequitable access to live-donor kidney transplantation.

**Strengths and limitations of this study**

- Pilot studies are a key phase of study development and design, and essential for evaluating any new research instrument and for informing the design of a full-scale study.
- The study questionnaire development has been described in detail, and the questionnaire evaluated in cognitive interviews.
- The findings of this pilot study will inform a sample size calculation for a full-scale study by providing data on frequency of exposures and variable distribution.
- As this is a pilot study, it is not designed to be powered to provide evidence for what factors explain the association between socioeconomic deprivation and reduced likelihood of live-donor kidney transplantation.

## Introduction

Live-donor kidney transplantation offers the best treatment in terms of life expectancy and quality of life^1–6^ for many patients with renal failure, and the possible long-term risks of live-donor nephrectomy are small.^7–11^ In the UK, there are no direct costs to an individual receiving a kidney transplant and potential donors are entitled to reimbursement from National Health Service (NHS) England, including for loss of earnings, travel costs and additional child care costs.^12^

Within the UK, socioeconomically deprived individuals are less likely to receive a live-donor kidney transplant (LDKT)^13^ ^14^ compared with less deprived individuals. The same has been demonstrated in the Netherlands,^15^ the USA^16–18^ and Australia.^19^ Little research exists on exploring the reasons for the observed inequity, and a US Consensus Conference in 2014 on Best Practices in Live Kidney Donation concluded that there is a real need to understand the mechanisms behind these observed disparities and identify targets for intervention.^20^

This study follows on from recently published qualitative work^21^ in which a series of semistructured interviews were undertaken with renal patients who had not received a LDKT. These interviews aimed to identify barriers to live-donor kidney transplantation, and compared the experiences of individuals from areas of high and low socioeconomic deprivation (SED). Four factors appeared to distinguish more deprived individuals from less deprived: (1) passivity, (2) disempowerment, (3) lack of social support and (4) short-term focus. In addition to a lack of social support, the emerging themes related to low levels of patient activation that is defined as an individual's knowledge, skill and confidence for managing their health and healthcare.^22^ ^23^ The qualitative interviews were analysed inductively and the work thus generated hypotheses that need further exploration. The questionnaire-based case–control study, of which this is a pilot, has been designed to further investigate and validate the qualitative themes, and to quantitatively examine the relationships between these variables and the type of transplant received. The qualitative study and subsequent questionnaire represent an ‘exploratory sequential mixed-methods design’^24^ in which elaboration, enhancement, illustration and clarification of the results of one method are sought with the results from the other method.^25^

The proposed full-scale study, of which this is a pilot, has been designed to assess whether SED is associated with:

1. The number of potential living kidney donors available to a transplant candidate, their suitability and the reasons any were considered unsuitable,
2. The social support experienced by a transplant candidate,
3. A transplant candidate's beliefs and knowledge about living kidney donation and transplantation, and
4. An individual's level of engagement in their healthcare.

The future full-scale case–control study will assess whether each of the variables above may be potential intermediaries in the causal pathway between SED and reduced odds of receiving a LDKT over a deceased-donor kidney transplant (DDKT). Thus, it will examine if the relationship between SED and transplant received is explained by differences in the variables listed above.

The study presented here is a pilot of the proposed case–control study, with the aim of estimating the following important components needed to design the future larger study:

1. Response rates;
2. Acceptability of the questionnaires;
3. Face validity of the content of the questionnaire;
4. Acceptance rates of linkage of questionnaire information to medical records;
5. Logistics, time and costs.

Findings from this study were required to inform a sample size calculation for a full-scale study by providing data on frequency of exposures, and variable distribution.

## Methods

This questionnaire-based pilot case–control study was a single-centre study. Participants were recruited from Southmead Hospital, North Bristol NHS Trust, and data analysis occurred at the School of Social and Community Medicine, University of Bristol.

### Questionnaire design

**Figure 1:**
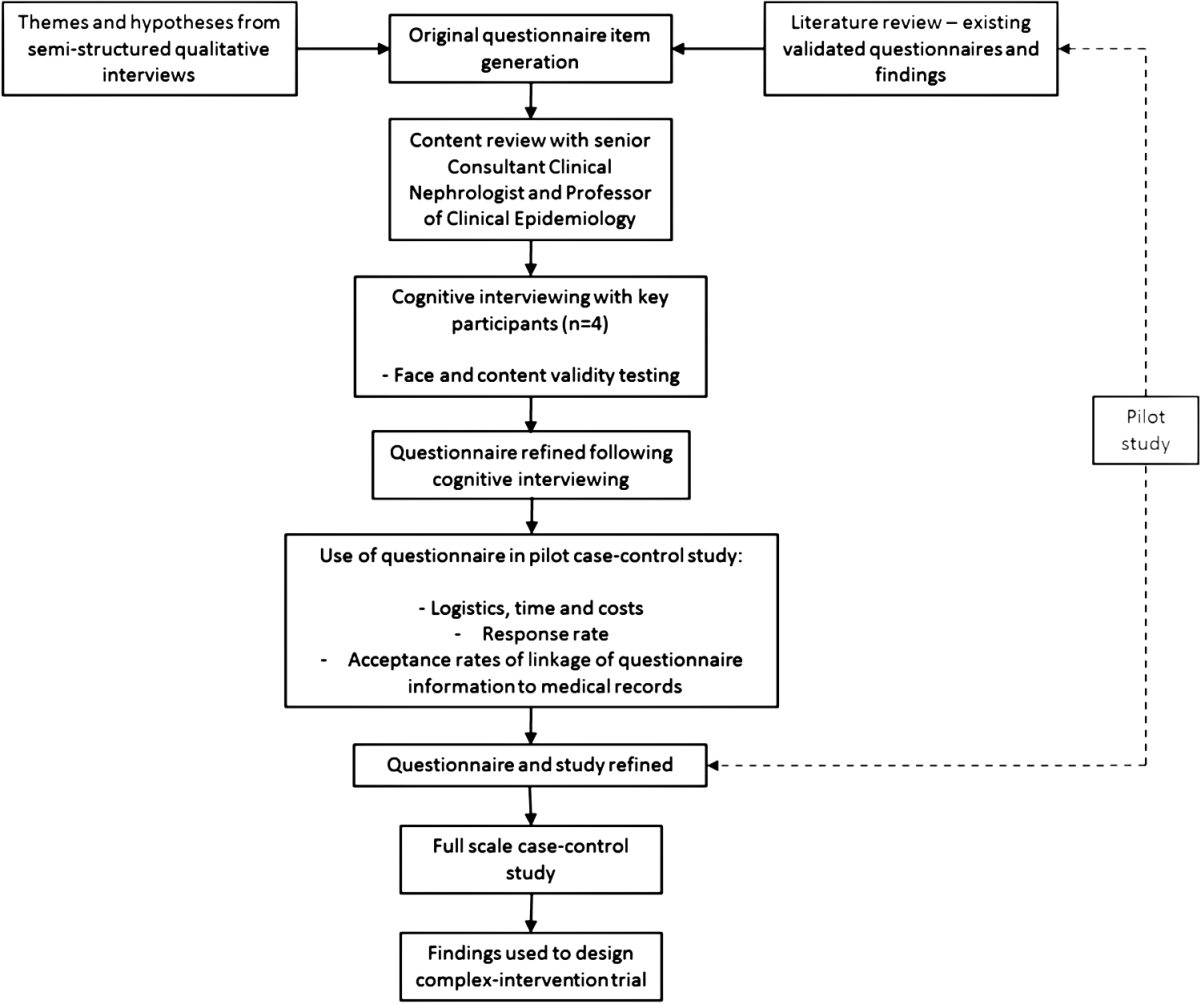
Research study and questionnaire development.

The overall study development is detailed in figure 1. The questionnaire was designed and refined between March 2015 and July 2015. Original item generation was informed by themes arising from qualitative research.^21^ A literature review was then undertaken to identify existing and validated questionnaires for exploring the topics we aimed to investigate. The use of existing questionnaires and validated measures allows for comparison between studies and populations, and enables possible meta-analysis to be performed.

### Questionnaire content

Individuals were asked how many potential donor relatives they had. They were asked whether any were considered suitable for donation, the reasons donors were not considered, whether any donors volunteered, whether any were asked to donate and whether any donors underwent assessment for donation.

Questionnaires also assessed participant demographics, renal replacement therapy (RRT) history and sources of health information. SED was evaluated at the individual level from education level, employment, income and housing tenure. It was also evaluated at the area level using a postcode-derived index of multiple deprivation (IMD) score.^26^ ^27^ The IMD measure is based on methodology developed at the University of Oxford Social Disadvantage Research Centre^26^ and is based on routine census data. Each country in the UK has individual components constituting an IMD score.^28^ There are seven domains of deprivation (income, employment, health and disability, education skills and training, barriers to housing and services, living environment deprivation, and crime) that determine the index score for an area in England, with higher scores indicating greater deprivation. IMD scores are nationally divided into five equal-sized population quintiles according to the level of deprivation of the output area to which these belong, with the fifth quintile representing the greatest deprivation. Therefore, the IMD area-level measure of SED was used as an ecological proxy for individual SED. Both area-level and individual-level socioeconomic variables were collected to allow for comparison of the two, as well as to assess the relationship between different aspects/components of SED and health behaviour and outcomes.

### Survey tools

The literature review identified the following survey tools which were incorporated into this study's questionnaire:

- Social support was measured using the Interpersonal Support Evaluation List (ISEL) shortened version 12-item survey.^29^ The 40-item ISEL^30^ is one of the most widely used instruments designed to assess perceived social support. The short form measure, ISEL-12,^29^ generates a total score that describes overall perceived social support, and three subscales representing perceived availability of appraisal (advice or guidance), belonging (empathy, acceptance, concern) and tangible (help or assistance, such as material or financial aid) social support.^29^ The ISEL-40 has shown good internal consistency, reliability, test–retest reliability, convergent validity,^30^ ^31^ and structural validity.^32^ The psychometric properties of the ISEL-12 have also been assessed and show good validity and reliability, including in populations similar to our study population in terms of age, ethnicity and gender.^33^ ^34^ Construct validity analyses have suggested that ISEL-12 scores are positively related to social network diversity, number of people in one's social network, and life engagement, and inversely related to perceived stress and negative affect.^34^
- Transplant knowledge was measured using the transplant section of the Rotterdam Renal Replacement Knowledge-Test (R3K-T).^35^ Prior to the development of this questionnaire, a validated and standardised test of knowledge about kidney disease and all treatment options was not available. This questionnaire was developed and validated in four groups: (1) patients on dialysis, (2) patients undergoing LDKTs, (3) the general population of the Netherlands and (4) the general population of North America. A psychometric analysis was performed using item response theory.^36^ This study resulted in a questionnaire, the R3K-T, which enables reliable testing of a patient's knowledge on kidney disease, and treatment options in clinic and research.^37^
- Transplant beliefs were assessed using questions from a questionnaire from research published by Stothers *et al*.^38^ ^39^ Although not formally validated, the questionnaires were reviewed by three expert focus groups that included physicians, surgeons, nurses and social workers, and piloted to test reliability. The authors report that test–retest analysis was performed, and there was no evidence of ‘skew’ or ‘halo’ effect on any subset of statements.^38^ No alternative questionnaire for use in this population group could be found.
- An individual's level of engagement in their healthcare was measured using Insignia Health's 13-point Patient Activation Measure (PAM).^22^ ^23^ ^40^ ‘Patient activation’ is a behavioural concept that incorporates the themes emergent from the qualitative work of passivity, disempowerment and limited knowledge. It is defined as ‘an individual's knowledge, skill and confidence for managing their health and healthcare’.^22^ ^23^ The PAM was originally developed as a 22-point scale and is validated and highly reliable.^41^ The 13-point shortened version was subsequently developed to be less burdensome and less costly to administer and complete. The psychometric properties of the shortened version have been assessed (and compared with the 22-point scale), and the measure has been shown to be valid and reliable.^22^ When the 13-item PAM score is regressed on the 22-item PAM score, it accounts for 92% of the variations in the 22-item version estimated activation. Construct validity assessment found that preventive behaviours, disease-specific self-management behaviours, and consumeristic behaviours are all strongly correlated with PAM-13 activation scores. The 13-item version has slightly lower reliability for some subgroups, including those with lower income and education, but these lower reliabilities are still within an acceptable range.^22^
- The PAM is a unidimensional, probabilistic Guttman-like scale^42^ that reflects a developmental model of activation. Patient activation appears to involve four stages: (1) believing the patient role is important, (2) having the confidence and knowledge necessary to take action, (3) actually taking action to maintain and improve one's health and (4) staying the course even under stress. The measure has good psychometric properties indicating that it can be used at the individual patient level to tailor intervention and assess changes.^41^ To calculate the total PAM score, the raw score is divided by the number of items answered (excepting non-applicable items) and multiplied by 13. Then, this score is transformed to a scale with a theoretical range 0–100, based on calibration tables, with higher PAM scores indicating higher patient activation. The raw scores can be converted into four activation levels: (1) (≤47.0) not believing activation is important, (2) (47.1–55.1) a lack of knowledge and confidence to take action, (3) (55.2–67.0) beginning to take action and (4) (≥67.1) taking action.

A range of different question response types were used in the questionnaire: numeric, multiple choice, true/false and scaled, by using 4-point and 10-point Likert items.^43^ Likert scaling is a bipolar scaling method which measures either a negative or positive response to a question or statement. The use of even number scales is sometimes described as a ‘forced choice’ method since the neutral option, which may be selected when a participant is unsure rather than truly neutral, is removed.

### Methods to encourage responses

In a Cochrane systematic review of methods to increase response rates to questionnaires,^44^ the following approaches were found to be successful, and for this reason were employed in designing the study questionnaire:

The questionnaire originated from a university (OR of university originated questionnaire vs other 1.32; 95% CI 1.13 to 1.54, I^2^ 83%).^44^ Questionnaires and invitation letters were both printed with the University of Bristol's logo.
The questionnaire content was of importance and interest to participants (OR 2.00; 95% CI 1.32 to 3.04, I^2^ 80%),^44^ as it explored a disease and treatments that the recipients all had experienced.
The questionnaire included an assurance of confidentiality (OR 1.33; 95% CI 1.24 to 1.42).^44^
The questionnaire was short (OR 1.64; 95% CI 1.43 to 1.87, I^2^ 91%).^44^ ^45^ The questionnaire was 14 A4 sides of size 12 font. The cognitive interviewees stated that they felt that the questionnaire was short.

### Cognitive interviewing

The cognitive interview is a method that can be used ‘to evaluate, and to improve, self-report survey questions, measurement instruments, research consent forms and other written materials’.^46^ The cognitive interview involves ‘the administration of draft survey questions while collecting additional verbal information about the survey responses, which is used to evaluate the quality of the response or to help determine whether the question is generating the information that its author intends’.^47^ Cognitive interviewing was thus undertaken to test each question for clarity, comprehension, face validity, sensitivity, acceptability, as well as the respondent's motivation to answer the question, the ease of retrieval of the required information and the suitability of response categories. A reparative approach was taken, which focused on improving the quality of survey questions and minimising the risk of response error.^46^ A summary of the cognitive interview findings is available as online supplementary material.

10.1136/bmjopen-2016-012132.supp1 Supplementary data

### Readability

The questionnaire had a Flesch^48^ reading ease measure^49^ of 68.3. The higher a Flesch rating, the easier the text is to understand. A score of 68.3 corresponds to text that is written in plain English, in which the average sentence is 15–20 words long, and the average word has two syllables. This reading ease level should be easily understood by students aged 13–15 years.

### Participant selection

Thirty LDKT recipients (cases) and 30 DDKT recipients (controls) were selected from all adults (age>18 years) with kidney transplanted at Southmead Hospital, North Bristol NHS Trust, between 1 August 2007 and 31 July 2013. Any individuals identified by their responsible clinician as lacking the mental capacity to consent to study participation according to the Mental Capacity Act 2005 were not eligible to participate. Random sampling of the eligible population, stratified by case–control status only, was used to select individuals for participation by using random numbers generated with Stata V.13 (StataCorp, Stata Statistical Software: Release 13 College Station, Texas, USA: StataCorp LP; 2013).

### Questionnaire distribution

Paper questionnaires were posted to participants in August 2015, which was accompanied by an invitation letter of one page in length,^50^ a Patient Information Sheet (PIS), and a stamped return envelope. The following approaches were employed to increase response rate, supported by evidence from a Cochrane systematic review:^44^

- The letters accompanying the questionnaire were personalised (OR of personalised letter vs non-personalised 1.14; 95% CI 1.07 to 1.22, I^2^ 63%).^44^
- Stamped return envelopes were used, as opposed to franked return envelopes (OR 1.24; 95% CI 1.14 to 1.35, I^2^ 69%).^44^
- Non-respondents were sent a second copy of the questionnaire (OR 1.41; 95% CI 1.02 to 1.94).^44^

In addition, the invitation letter was signed using a scanned signature by the potential participant's consultant nephrologist. Weak evidence suggests this may increase the likelihood of response (OR for more senior or well-known person vs less 1.13; 95% CI 0.95 to 1.35).^51^ Brown envelopes were used (OR brown vs white 1.52; 95% CI 0.67 to 3.44),^51^ ^52^ and the primary investigator's contact details were provided in case of any questions.^53^

Non-responders were sent a second questionnaire after 4 weeks. A consent form formed the first page of the questionnaire, including the request to link the questionnaire information to information in the participant's renal medical records (eg, postcode in order to derive an IMD score, renal diagnosis and history of RRT).

### Data collection

Study data were collected and managed using Research Electronic Data Capture (REDCap) electronic data capture tools hosted at the University of Bristol.^54^ REDCap is a secure, web-based application designed to support data capture for research studies.

### Data analysis and statistical methods

Findings from this study were required to inform a sample size calculation for a full-scale study by providing data on frequency of exposures and variable distribution. Basic statistical analysis was performed to explore the associations between the potential intermediaries (eg, size of potential donor pool, level of social support, level of patient activation) and case–control (transplant type) status by simple cross-tabulations and mean differences. Medians and IQRs were calculated for continuous variables. χ^2^, Fisher's exact and a non-parametric k-sample tests on the equality of medians were used to compare baseline characteristics.

The statistical analysis planned for the full-scale study will more formally quantify the associations using multivariable logistic regression analysis (OR, 95% CI, p value). The full-scale study will examine how SED, either as an individual or composite variable, predicts case–control status. Using mediation analysis, we will test whether conditioning on the intermediaries attenuates the observed association between SED and case–control status either partially or completely using multivariable logistic regression. In addition, we will also test for interactions between SED and the exposure variables to examine if the relationship between the potential intermediaries and outcome varies by SED in the larger study.

## Results

Questionnaires were posted to 60 potential study participants. Sixty-three per cent (n=38) of those invited returned questionnaires. Thirty-five individuals responded to the first questionnaire, and three responded to reminders. Seventy per cent (n=21) of invited LDKT recipients and 57% (n=17) of invited DDKT recipients responded (table 1). Reasons for non-response were not explored for ethical reasons, but non-respondent characteristics were analysed. Along with DDKT recipients, men, individuals aged 20–39 years, and individuals from areas in IMD quintiles 4 and 5 were less likely to respond (table 2). Every participant consented to linkage of the questionnaire data to information from their medical records.

**Table 1:** Participant characteristics

	Cases Live-donor kidney transplant recipients n=21	Controls Deceased-donor kidney transplant recipients n=17
Sex (%)		
Women	12 (57)	11 (65)
Men	9 (43)	6 (35)
Age category in years (%)		
20–39	5 (24)	1 (6)
40–59	5 (24)	11 (65)
60–79	11 (52)	5 (29)
Ethnicity (%)		
White	18 (86)	15 (88)
Other	3 (14)	1 (6)
IMD quintile (%)		
1 (least deprived)	6 (29)	6 (35)
2	3 (14)	6 (35)
3	7 (33)	4 (24)
4	4 (19)	0
5 (most deprived)	1 (5)	1 (6)
Mean number of potential family donors (SD)	14 (12)	14 (12)
Median number of potential family donors (IQR)	11 (13)	11 (7)
Mean PAM score (SD)	64.2 (12.4)	64.5 (15.5)
Median PAM score (IQR)	67.8 (19.4)	60.6 (24.1)
Per cent of PAM level 4 (95% CI)	38 (15 to 61)	35 (10 to 61)
Mean social support score (SD)	30.0 (6.0)	29.6 (5.3)
Median social support score (IQR)	31.0 (7.0)	30.5 (9.0)
Mean live-donation transplant knowledge score (SD)	6.7 (SD 1.8)	4.9 (SD 2.1)
Median live-donation transplant knowledge score (IQR)	7.0 (2.0)	5.0 (2.0)

IMD, index of multiple deprivation; PAM, Patient Activation Measure.

**Table 2:** Participants and non-participants

	Participants n=38 (% invited)	Non-participants n=22 (% invited)
Sex		
Women	23 (77)	7 (23)
Men	15 (50)	15 (50)
Age category in years (%)		
20–39	6 (43)	8 (57)
40–59	16 (62)	10 (38)
60–79	16 (80)	4 (2)
Ethnicity		
White	34 (62)	21 (38)
Other	4 (80)	1 (20)
Renal transplant type		
Live-donor	21 (70)	9 (30)
Deceased-donor	17 (57)	13 (43)
IMD quintile		
1 (least deprived)	12 (67)	6 (33)
2	9 (75)	3 (25)
3	11 (61)	7 (39)
4	4 (57)	3 (43)
5 (most deprived)	2 (40)	3 (60)

IMD, index of multiple deprivation.

### Missing data

One hundred per cent of the questions on transplant knowledge, transplant beliefs, social support and patient activation were completed. Three of the nine individuals who had received pre-emptive transplants, and therefore had not received any form of dialysis, did not answer the questions on preparation for dialysis, which suggests that dialysis options were not considered for back-up if the transplant did not go ahead, as suggested by one of the cognitive interviewees.

There was a large amount of missing data for the question on income, suggesting that these questions were less acceptable to participants. This was also highlighted in the cognitive interviewing. Sixteen (42%) selected ‘would rather not answer’ on the income question. These participants represented all IMD quintiles, so it cannot be assumed non-disclosure represents a low or a high income. Three individuals declined to disclose their education history, two preferred not to disclose their employment status and one chose not to disclose their housing tenure status.

### Transplant preference

Participants were asked to recall if they had a preference for a certain kidney transplant type prior to receiving a transplant. Prior to transplantation, the preferences of eventual LDKT and DDKT recipients differed significantly (p=0.05). Fifty-seven per cent of eventual LDKT recipients stated that a living-donor transplant was their preferred transplant type, with the majority favouring a ‘known donor’, as compared with 18% of eventual DDKT recipients. The majority (65%) of eventual DDKT recipients expressed that they had had no preference between a DDKT and a LDKT. After transplantation, the preferences of LDKT and DDKT recipients remained significantly different (p=0.02). Sixty-two per cent of LDKT recipients continued to prefer a LDKT as compared with 6% of DDKT recipients. The majority (71%) of DDKT recipients still expressed no preference between LDKT and DDKT after transplantation.

The most useful sources of information on RRT were reported as discussions with a healthcare professional (97% of participants selected), and written information provided by a hospital (55% of participants selected).

The mean number of potential related donors available to an individual did not differ by transplant type (table 1), but there was a suggestion of variation with SED (table 3). More deprived transplant recipients appeared to report smaller numbers of potential donors than less deprived transplant recipients. This trend appeared to be statistically significant for cases (p=0.05). Fifty-eight per cent of participants had not asked any of their potential donors to consider living kidney donation (52% LDKT vs 65% DDKT, p=0.44).

**Table 3:** Median number of potential family donors stratified by socioeconomic deprivation and transplant type

IMD quintile	Cases Live-donor kidney transplant recipients	Controls Deceased-donor kidney transplant recipients
	Median number of potential family donors	Median number of potential family donors
1 (least deprived)	21	13
2	11	11
3	7	11
4	10	–
5 (most deprived)	7	8

IMD, index of multiple deprivation.

### Live-donor kidney transplantation knowledge and beliefs

There was some evidence of a difference between the R3K-T knowledge score for recipients of LDKTs (mean 6.7, SD 1.8), and for recipients of DDKTs (mean 4.9, SD 2.1; p=0.008).

The degree of agreement with various belief statements by case–control status is presented in table 4. With the small pilot sample size, only agreement with one statement differed significantly between LDKT and DDKT recipients. In total, 76.2% of LDKT recipients agreed and 14.3% strongly agreed with the statement ‘a LDKT may strengthen the relationships between the donor and recipient’. This compared with 41.2% of DDKT recipients agreeing with this statement, with 35.3% being uncertain (p=0.03).

**Table 4:** Beliefs about living donation and live-donor kidney transplantation

Belief statement	Transplant type	Strongly disagree (%)	Disagree (%)	Agree (%)	Strongly agree (%)	Don't know (%)	χ^2^ p Value
It is ethically acceptable to take a kidney from a healthy person.	L	4.8	4.8	61.9	28.6	0	0.56
	D	0	5.9	47.1	47.1	0	
Donors often agree to donate due to feelings of guilt or family pressure.	L	14.3	38.1	14.3	4.8	28.6	0.61
	D	0	47.1	17.7	5.9	29.4	
Donating a kidney is a rewarding experience for the live donors.	L	0	0	66.7	23.8	9.5	0.49
	D	0	0	58.8	17.7	23.5	
Donating a kidney to someone requires an extremely close personal relationship.	L	4.8	61.9	14.3	14.3	4.8	0.50
	D	5.9	35.3	23.5	17.7	17.7	
A live donor kidney transplant may strengthen the relationships between the donor and recipient.	L	4.8	4.8	76.2	14.3	0	**0.03**
	D	5.9	11.8	41.2	5.9	35.3	
Approaching a potential donor who then says no will change the relationships between the two people.	L	9.5	23.8	33.3	4.8	28.6	0.21
	D	5.9	58.8	11.8	0	23.5	
Asking someone to donate makes the recipient seem selfish or greedy.	L	0	45.0	25.0	20.0	10.0	0.40
	D	0	35.3	35.3	5.9	23.5	
It is acceptable for a parent to receive a kidney from his/her child (over 18 years old).	L	0	4.8	81.0	9.5	4.8	0.25
	D	5.9	11.8	47.1	17.7	17.7	
Decisions about donation should be made by the donor alone. The recipient should not ask for a kidney.	L	4.8	28.6	33.3	28.6	4.8	0.54
	D	0	29.4	47.1	11.8	11.8	
Since the donor operation is not risk free, someone who needs a kidney transplant should wait for a kidney from someone who has died.	L	23.8	66.7	0	0	9.5	0.15
	D	5.9	58.8	0	5.9	29.4	

Bold indicates p value <0.05.

D, deceased-donor; L, live-donor.

### Patient activation and social support

A two-sided t-test was conducted to compare the degree of patient activation, social support and living kidney donation knowledge of LDKT and DDKT recipients. There was no evidence of a difference between PAM-13 score for recipients of LDKTs (mean 64.2, SD 12.4) and recipients of DDKTs (mean 64.5, SD 15.5; p=0.94). There was no evidence of a difference between the ISEL-12 social support score for LDKT recipients (mean 30.0, SD 6.0) and DDKT recipients (mean 29.6, SD 5.3; p=0.84).

### Logistic regression analysis

The logistic regression analysis was largely performed to demonstrate the planned analysis for the larger study; the findings are presented in table 5 for illustrative purposes only. The area-level measure of SED, the IMD score, was compared with individual-level measures of SED, including income, education and employment level. With the pilot data, findings were not statistically significant, apart from the model exploring the relationship between income and likelihood of LDKT; in the unadjusted model, increasing income was associated with a greater likelihood of having had a LDKT (OR 1.98, 95% CI 1.12 to 3.50, p=0.02). In the fully adjusted model, this relationship persisted (OR 2.15, 95% CI 1.13 to 4.08, p=0.02). As expected, the IMD score showed a weaker association in the unadjusted model but after adjustment, the OR was similar to that of the employment variable.

**Table 5:** ORs for the association between being a live-donor kidney transplant recipient and socioeconomic deprivation

Measure of socioeconomic deprivation	Unadjusted model OR (95% CI)	Age, sex and ethnicity adjusted model OR (95% CI)
IMD quintile* (n=38)	1.48 (0.82 to 2.64) p=0.19	1.82 (0.90 to 3.69) p=0.10
Income† (n=22)	1.98 (1.12 to 3.50) p=0.02	2.15 (1.13 to 4.08) p=0.02
Level of education‡ (n=34)	2.16 (0.98 to 4.76) p=0.06	2.05 (0.88 to 4.79) p=0.10
Level of employment§ (n=20)	1.65 (0.75 to 3.62) p=0.21	1.75 (0.70 to 4.34) p=0.23

*OR per increase in IMD quintile.

†OR per £500 increase in monthly household income.

‡OR per increase in education level: no formal education/training; primary school; secondary school; vocational/technical/trade training; university undergraduate degree; university postgraduate degree.

§OR per increase in level of unemployment (excluding retirees, homemakers and full-time education).

IMD, index of multiple deprivation.

## Discussion

This study was a pilot of a case–control study which has estimated the important parameters needed to design a future larger main study.

### Response rates

The response rate of 63% overall and 70% of invited LDKT recipients is similar to the response rate reported in a published survey of transplant recipients in France (60% response rate).^55^ The response rate of 70% from invited LDKT recipients is similar to that of 70.4% of LDKT recipients in the Netherlands who were evaluated using the R3K-T questionnaire.^35^

The second mail-out generated 8% of the overall responses and will, therefore, be repeated in the full-scale study. To further increase response rate, at minimal additional cost, electronic reminders will be sent via text message or email.^56^ The full-scale study will be based at multiple centres, some of which will serve populations with greater levels of SED than the population served by North Bristol NHS Trust. This should help to increase responses from renal patients in IMD quintiles 4 and 5.

### Acceptability of the questionnaire and data linkage

Cognitive interviewing confirmed that the questionnaires were acceptable, with possibly sensitive personal and socioeconomic questions highlighted. Cognitive interviewees suggested that the option of ‘would rather not answer’ be included for these questions. The good response rate and the overall lack of missing data both suggest that the questionnaires were acceptable.

Almost half of the participants indicated that they would rather not answer a question on income. However, the majority were willing to answer other socioeconomic questions, including education, employment status, and property ownership. All participants consented to the linkage of the questionnaire to data stored in the medical records; therefore, IMD scores could be derived from postcode to generate an area-level measure of the SED experienced by a participant.

### Face validity of the content of the questionnaire

The questionnaire comprised a number of previously validated scales and assessment tools. The findings of this pilot are in agreement with existing literature: the R3K-T live donation questionnaire norm-reference score in LDKT recipients has been reported as 6.89 (2.48),^37^ compared with 6.7 in our LDKT cases. This suggests that questions were understood as in previous studies.

Cognitive interviewing confirmed that the questions were transparent and interpreted as intended; thus, the questionnaire items had good face validity. The low rate of missing data suggests that questions were apparently understood, with the exception of the questions on dialysis decision-making. It was assumed when developing the questionnaire that all individuals would have to some extent consider all RRT options, even if they had planned for and received a pre-emptive transplant. Most recipients of pre-emptive transplants indicated that they had indeed considered dialysis options, but a few individuals indicated that they had not considered dialysis at all. A filter question will be introduced to the questionnaire in the full-scale study asking if participants ever considered dialysis.

### Limitations

Pilot studies are a key phase of study development and design, and essential for evaluating any new research instrument and for informing the design of a full-scale study. The study questionnaire development has been described in detail and the questionnaire evaluated in cognitive interviews. The findings of this pilot study will inform a sample size calculation for a full-scale study by providing data on frequency of exposures and variable distribution. However, this study has some limitations. First, the questionnaire was piloted in a transplant centre that serves a predominantly white British population, and all invited participants spoke English. Although the questionnaire will be translated, if required, in the full-scale study, the response rate from individuals whose first language is not English may differ to that from those in this study. A second limitation of this study is that the questionnaire is to be administered to LDKT and DDKT transplant recipients, both of whom have experienced transplantation, and thus any detected differences in beliefs and knowledge may reflect this experience and be subject to a range of cognitive biases, including justifying their decision, recall bias and endowment effects. How important these biases might be for the interpretation of findings depends on whether a difference is detected in the full-scale study.

### Future research: sample size calculation

The questionnaire has been designed to measure multiple factors, as detailed above, but we have decided to power a future study on the patient activation variable.

Based on the distribution of previously observed scores, the ‘½ SD’ estimate^57^ has been suggested as approximating a minimal important difference (MID) for patient-reported instruments.^57^ ^58^ Empirical evidence from previous studies, physiological arguments and statistical theory show a tendency to converge to the ½ SD criteria as being meaningful to patients.^59^

Using this distribution-based method to estimate a clinically meaningful MID, the pilot study overall mean PAM-13 score's SD was 14; therefore, a seven-point difference between cases and controls would be meaningful to detect. This SD is comparable to previously published studies of patient activation,^60^ ^61^ including those of patients with chronic kidney disease stages 4 and 5.^62^ The mean PAM-13 score in our pilot study LDKT cases was 64.2.

To detect a difference of seven points (½ SD) between LDKT cases and DDKT controls (ie, 64 vs 57) in IMD quintile 5 at 90% power, 5% significance and a 1:1 ratio would require 85 participants per group (170 total). Therefore, to be able to test for a ½ SD difference across all five quintiles, we need a sample size of 850. We anticipate there were some missing data. Therefore, in the final complete case analysis, we may have a reduction of 10% thereby necessitating 944 participants. In addition, the response ‘rate’ for the pilot study was 63% (n=38/60), thereby requiring an initial sample size of 1500 participants (750 cases, 750 controls) to be approached and hence, the need for four centres as a minimum. We can detect a far smaller difference (0.16 SD) for a dichotomous exposure or between 6% and 8% for a categorical outcome.

In order to recruit this study population from individuals with kidneys transplanted between 2010 and 2015, the full-scale study will have to run at four transplant centres.^63^ This pilot-informed sample size calculation will also allow for accurate costing of a full-scale study.

### Future statistical analysis plan

In the full-scale study, we will initially explore how SED, either as an individual or composite variable, predicts case–control status. We will then test if our potential intermediaries (eg, size of potential donor pool, level of patient activation) are themselves predicted by case–control status by using simple cross-tabulations and mean differences. More formal quantification will be undertaken using logistic regression analysis (OR 95% CI, p value). We will then use mediation analysis to test whether conditioning on the intermediaries attenuates the observed association between SED and case–control status, either partially or completely, using multivariable logistic regression. We will also test for interactions between SED and the exposure variables to examine if the relationship between the potential intermediaries and outcome varies by SED. For example, we will explore whether poor social support is more important in more deprived patients than less deprived for explaining case–control status.

### Future interventions

This pilot study will enable us to run a successful full-scale questionnaire-based kidney transplant case–control study, the findings of which will help us better understand how SED is related to the type of transplant an individual receives. This understanding will help us to design and appropriately tailor an intervention to reduce inequitable access to live-donor kidney transplantation. If the socioeconomic inequity in live-donor kidney transplantation is found to be associated with lack of social support, not asking for potential donors, low levels of patient activation, or a lack of knowledge then a number of possible interventions exist that could be targeted at those most likely to benefit. These include the use of transplant candidate advocates^64^ who are individuals who advocate for a renal patient suitable for transplantation, discussing living donation with potential donors on the transplant candidate's behalf. Transplant candidate advocates may mean that people who a transplant candidate had perceived as not offering social support and not being willing to donate actually do come forward to undergo evaluation. Patient empowerment interventions designed to increase patient activation include tailored coaching,^65^ counselling with information sheets^66^ ^67^ and support for preparing questions for a consultation.^68^ The findings of the full-scale study are essential to ensure the right intervention is developed and trialled, and targeted at those most likely to benefit.
