# Supplementary material for: What factors explain the association between socioeconomic deprivation and reduced likelihood of live-donor kidney transplantation? A questionnaire-based pilot case-control study
Source: BMJ Open. 2016 Jun 9;6(6):e012132. doi: 10.1136/bmjopen-2016-012132 (PMC4908905; doi:10.1136/bmjopen-2016-012132)
Supplement: Supplementary data [file bmjopen-2016-012132supp.pdf]

Supplementary material – Summary of cognitive interview findings

Four individuals were interviewed, purposefully selected to differ in age, sex, and socioeconomic position (Table 1). Three interviews were conducted in person, whilst one was conducted over the telephone. Individuals were asked to read through the questionnaire whilst thinking aloud, informing the researcher of their opinions on the clarity, intelligibility and simplicity of the question, as well as the ease with which the question could be answered and the suitability of the response options. At the end participants were asked to comment on the overall appearance, layout and length of the questionnaire. The participant and researcher (PB) each had a copy of the questionnaire. Notes were taken by the researcher while the participant worked through the questionnaire.

Table 1: Characteristics of cognitive interviewees

	Cognitive interviewee			
	1	2	3	4
**Sex**	Male	Male	Female	Female
**Age group (years)**	60-69 years	30-39 years	60-69 years	30-39 years
**Index of Multiple Deprivation (IMD) 2010 decile**	10	1	6	7

Across the four interviews the types of dialysis were not universally understood. One participant did not know that there was more than one type of dialysis and required more information to ascertain which type he had received. A simple description of the types of dialysis was therefore added to questions where appropriate.

One participant advised that because they had received a pre-emptive kidney transplant they had not considered dialysis options, and were therefore unable to answer questions on this. After discussion with other participants it was felt that most people should have considered dialysis options even if they planned and received a pre-emptive transplant, and therefore these questions remained.

Regarding the question on potential donors, two interviewees advised that some participants might not know the number of siblings or cousins they have, especially in the context of family breakdown. It was therefore suggested that the option of ‘I don’t know’ was added to this question.

One participant stated that the question ‘How many people from the previous table have you communicated with in the last year?’ was a difficult question to answer, and asked what the point of the question was. After discussion with the researcher it became clear that the question was aiming to assess the closeness of relationships, but this was assessed separately, and more explicitly, in another question. Therefore the question on communication was removed.

Two participants advised that the section on social support required a time focus. One asked ‘When is this for? Now or when I was getting my transplant?’ Therefore a sentence was added to this section advising that ‘When you are answering these questions, please think about your current situation.’ This section contained potentially sensitive questions and the researcher specifically explored the acceptability of these questions with participants. All felt that the questions were not too sensitive for them, but suggested that a sentence specifying that a question could be left blank would be a reasonable addition. The addition

of contact details of the researcher if the participants felt they wished to discuss this further was also suggested.

One interviewee pointed out that one question extracted from a previously published questionnaire (1) referred to a ‘cadaver donor kidney’ and that this term is no longer acceptable. The term was changed to ‘a kidney from someone who has died’.

Finally it was suggested by one participant that all the questions in the section on demographics should include a ‘Would rather not answer’ response option.

References

1. Stothers L, Gourlay W, Liu L. Attitudes and predictive factors for live kidney donation: a comparison of live kidney donors versus nondonors. Kidney Int 2005 Mar;67(3):1105-11.
